
JOURNAL INFORMATION
==============================
NLM Title Abbreviation: Innov Surg Sci
Journal ID: Innov Surg Sci
NLM Journal ID: 101708165
Journal ID: iss

Innovative Surgical Sciences

EISSN: 2364-7485
Publisher: De Gruyter

ARTICLE INFORMATION
==============================
PMCID: PMC10216886
DOI: 10.1515/iss-2023-9005
Article ID: iss-2023-9005
Article version: 1
Subjects: Article

Abstract DGOOC

Publication date: 2023 Mar
Electronic publication date: 2023 Mar 13
Volume: 8
Issue: Suppl1
First page: 123
Last page: 128
Copyright: © the Author(s), published by De Gruyter
Copyright year: 2023
Copyright holder: the Author(s), published by De Gruyter
License: This work is licensed under the Creative Commons Attribution 4.0 International License.
License URL: https://creativecommons.org/licenses/by/4.0/

Page count: 6

==============================

**Advantages and risks of wound drain in tumor spine surgery: A multicenter surveillance study of 10,029 patients from the German Spine Registry (DWG-Register)**

ID: 275

**Kategorie: DGOOC - Muskuloskeletale Tumoren – interdisziplinäre Versorgung**

Sebastian Walter[1], Maximilian Lenz[1], Peter Knöll[1], Kourosh Zarghooni[1]

[1]*Universitätsklinikum Köln (AÖR), Klinik für Orthopädie und Unfallchirurgie, Köln, Deutschland*

**Background:**

There is an ongoing debate whether a surgical drain is beneficial to prevent local accumulation of hematoma and to reduce the rate of wound infections, and neurological deficits.

**Materials and methods:**

Data from the German Spine Society (DWG) registry were filtered for surgically treated spine tumor cases between 2017 and 2021. Cases were categorized into such with (Group I) and without (Group II) placement of a surgical drain. Subgroups were compared for demographic data, type of surgery, experience of the surgeon and postoperative surgical complications.

**Results:**

10,029 cases were included into final analysis (Group I: 3,007; Group II: 7,022). There was no significant difference between both groups regarding age or gender distribution. Average morbidity of patients was significantly elevated in Group I ($p < 0.05$) and the rates of invasive surgery were significantly increased in this group ($p < 0.001$). Overall complication rates were reported with 12.0% (Group I) and 8.5% (Group II). There were significantly more epidural hematoma ($p < 0.001$) and motor dysfunction ($p = 0.049$) as well as deep wound infections ($p < 0.001$) in Group I.

**Conclusion:**

A surgical drain cannot prevent epidural hematoma.

Open Access. © 2023 The Author(s), published by De Gruyter. This work is licensed under the Creative Commons Attribution 4.0 International License.

**Predictors for quality of life, pain and functional outcomes after surgical treatment of metastatic disease in the spine**

ID: 277

**Kategorie: DGOOC - Muskuloskeletale Tumoren – interdisziplinäre Versorgung**

Sebastian Walter[1], Jan Hockmann[1], Maximilian Weber[1], Kourosh Zarghooni[1]

[1]*Universitätsklinikum Köln (AÖR), Klinik für Orthopädie und Unfallchirurgie, Köln, Deutschland*

**Background:**

While predictors for postoperative survival in spine tumor patients have been identified, there is limited evidence for predictors of postoperative Quality of Life (QoL), pain and functional outcome.

**Materials and methods:**

153 consecutive patients who underwent surgery for symptomatic spinal metastases between June 2016 and April 2019 were interviewed preoperatively and for follow-up at three, six and twelve months using the EQ-5D-3L, COMI, and ODI questionnaires. Differences in means exceeding specific Minimal Clinically Important Difference (MCID) values were indicated as clinically significant.

**Results:**

33% of the patients had been reported dead after twelve months. Only one metastasis multiplies by 7.9 the probability for an improved EQ-5D-3L score at three months compared to multiple metastases. No neoadjuvant metastatic irradiation multiplies this probability by 6.8 against performed radiation. A preoperative ODI score between 50.1 and 100 has 22.0 times the odds compared to the range from 0 to 50 for an improved EQ-5D-3L after three months, and 12.5 times the odds in favor of improved COMI after three months and 13.6 times the odds for improvement of ODI at three-month follow-up. A preoperative COMI score ranging from 5.0 to 10 has 21 times the odds of a COMI between 0 to 5 for an improved EQ-5D-3L score and 11 times the odds for an improved ODI after twelve months. Other suspected predictors showed no statistically significant improvement.

**Conclusion:**

An improvement of QoL, pain and spinal function after twelve months can be predicted by a subjective preoperative poor health condition. Impaired spinal function before surgery, a singular metastasis and no previous irradiation is predictive of improved spinal function and quality of live three months after surgery.

**Surgical treatment principles for soft tissue sarcomas: consensus of the surgical societies based on the S3 guideline.**

ID: 287

**Kategorie: DGOOC - Muskuloskeletale Tumoren – interdisziplinäre Versorgung**

Jens Jakob[1], Dimosthenis Andreou[2], Benedicta Beck-Broichsitter[3], Jens Bedke[4], Wilfried Budach[5], Dominik Denschlag[6], Hans-Roland Dürr[7], Moritz Felcht[8], Markus Follmann[9], Steffen Frese[10], Thomas Gösling[11], Thomas Graeter[12], Viktor Gruenwald[13], Robert Gruetzmann[14], Jürgen Hoffmann[15], Peter Hohenberger[1], Vlada Kogosov[16], Wolfram Trudo Knoefel[17], Marcus Lehnhardt[18], Burkhard Lehner[19], Lars H. Lindner[20], Cordula Matthies[21], Peter Reichardt[22], Jalid Sehouli[23], Selma Ugurel[24], Bernd Kasper[25]

[1]*Universitätsmedizin Mannheim, Medizinische Fakultät Mannheim der Universität Heidelberg, Chirurgische Klinik, Mannheim, Deutschland*, [2]*Medizinische Universität Graz, Universitätsklinik für Orthopädie und Traumatologie, Graz, Österreich*, [3]*Klinikum Stuttgart, Klinik für Mund-, Kiefer- und Gesichtschirurgie, Stuttgart, Deutschland*, [4]*Universitätsklinikum Tübingen, Universitätsklinik für Urologie, Tübingen, Deutschland*, [5]*Universitätsklininkum Düsseldorf, Klinik für Strahlentherapie und Radioonkologie*, [6]*Hochtaunus Kliniken, Gynäkologie*, [7]*Ludwig Maximilian Universität München, Muskuloskelettales Universitätszentrum München*, [8]*St. Josefs Krankenhaus , Zentrum für Dermatochirurgie, Heidelberg, Deutschland*, [9]*Deutsche Krebsgesellschaft, Leitlinienprogramm, Berlin, Deutschland*, [10]*Lungenklinik Lostau, Klinik für Thoraxchirurgie, Lostau, Deutschland*, [11]*Städtisches Klinikum Braunschweig, Unfallchirurgie, Braunschweig, Deutschland*, [12]*Fachklinik Löwenstein, Klinik für Thorax- und Gefäßchirurgie, Löwenstein, Deutschland*, [13]*Universitätsklinikum Essen, Interdisziplinäre Uroonkologie, Essen, Deutschland*, [14]*Uniklinikum Erlangen, Chirurgische Klinik, Erlangen, Deutschland*, [15]*Universitätsklinikum Heidelberg, Klinik und Poliklinik für Mund-, Kiefer- und Gesichtschirurgie, Heidelberg, Deutschland*, [16]*Universitätsmedizin Göttingen, Universitätskrebszentrum, CCC-N, Göttingen, Deutschland*, [17]*Universitätsklinikum Düsseldorf, Klinik für Allgemein-, Viszeral- und Kinderchirurgie, Düsseldorf, Deutschland*, [18]*BG Universitätsklinikum Bergmannsheil Bochum, Plastische Chirurgie und plastisch-rekonstruktive Chirurgie, Bochum, Deutschland*, [19]*Universitätsklinik Heidelberg, Klinik für Orthopädie, Heidelberg, Deutschland*, [20]*LMU Klinikum München, Medizinische Klinik und Poliklinik III, München, Deutschland*, [21]*Universitätsklinikum Würzburg, Neurochirurgische Klinik und Poliklinik, Würzburg, Deutschland*, [22]*Helios Klinikum Berlin Buch, Onkologie und Palliativmedizin, Berlin, Deutschland*, [23]*Charité - Universitätsmedizin Berlin, Klinik für Gynäkologie, Berlin, Deutschland*, [24]*Universitätsklinikum Essen, Klinik für Dermatologie, Essen, Deutschland*, [25]*Universitätsmedizin Mannheim, Medizinische Fakultät Mannheim der Universität Heidelberg, Mannheim Cancer Center, Mannheim, Deutschland*

**Background:**

In September 2021, the first evidence-based S3 guideline "Adult Soft Tissue Sarcomas" was published (AWMF registry no. 032/044OL). The guideline was funded by the German Cancer Aid within the German Guideline Program in Oncology. Principles of sarcoma treatment including recommendations about preoperative assessment, surgery and multimodal treatment were defined. Since soft tissue sarcoma may occur at any localization, representatives of all surgical societies were involved in the S3 guideline group. Here, we present the 10 most important recommendations for surgeons as selected by representatives of the surgical societies and the members of the S3 guideline working group "Therapy of Localized Soft Tissue Tumor".

**Materials and methods:**
Each representative of the surgical societies and each member of the guideline working group "Therapy of Localized Soft Tissue Tumor" was contacted to select the 15 most important recommendations. Votes for similar recommendations were summed (e.g., 5.20.: "In the absence of evidence of lymphogenic metastasis, lymphadenectomy should not be performed as part of primary tumor resection." and 5.22.: "In cutaneous sarcomas, sentinel lymph node biopsy (SLNB) should not be performed."). From the resulting ranked list, the 10 most frequently chosen recommendations were selected and confirmed by consensus.

**Results:**
The expert representatives selected the following 10 recommendations from the S3 guideline indicated by rank order: 5.6., 4.7., 5.15., 5.5., 4.15., 5.2., 5.20., 4.12., 4.3., and 5.16. The most frequently selected was the evidence based statement 5.6.: "Resection of primary soft tissue sarcoma of the extremities should be performed as a wide resection. The goal is an R0 resection." Other recommendations included the need for preoperative biopsy (4.7.), the recommendation to perform excisional biopsy only for superficial tumors up to 3 cm in size (4.12.), to perform a preoperative MRI imaging with contrast media (4.3.), and to discuss sarcoma cases preoperatively in a multidisciplinary sarcoma board (5.2.).

**Conclusion:**
The evidence-based S3 guideline "Adult Soft Tissue Sarcomas" is a milestone to improve the care of sarcoma patients in Germany. The selection of the top ten recommendations by surgeons for surgeons has the potential to improve the dissemination and acceptance of the guideline and to strengthen the constructive discussion about diagnostic and treatment strategies among the different disciplines.

**Indocyanine Green (ICG) Coloring of the Sciatic ,Common Peroneal and Tibial Nerves for Rotationplasty Surgery of Osteosarcoma Patients**
ID: 587

**Kategorie: DGOOC - Muskuloskeletale Tumoren – interdisziplinäre Versorgung**

Eric Pion[1], Thiha Aung[1,2]

[1]*Lehrstuhl für Molekulare und Zelluläre Anatomie, Regensburg, Deutschland*, [2]*TH Deggendorf, Deggendorf, Deutschland*

**Background:**
Sarcomas are highly aggressive tumors and often located in the extremities. Especially osteosarcomas mostly affect children and adolescence and several biological reconstruction methods have been developed for the reconstruction of infiltrated extremities. One being the Van Nes-Borggreve rotationplasty that avoids amputation even in severe cases in which the tumor affects the distal femur or proximal tibia. During this reconstructive procedure, the preservation of the sciatic nerve and its branches, the tibial and peroneal nerves, is of upmost importance for the muscle innervation. In this case series, we propose the epineural application of indocyanine green for the visualization of nerves in order to perform a safe tumor resection and to achieve the preservation of the extremity with a good functional outcome.

**Materials and methods:**
Three patients aged seven, nine and twelve years who were diagnosed with osteosarcoma of the proximal tibia or distal femur were selected. Prior to tumor resection, an epineural injection of indocyanine green was performed to highlight the epineural tissue in order to avoid surgical damage and to evade an epineurectomy.

**Results:**
Successful tumor resection and subsequent rotationplasty was performed with no damage to the sciatic, tibial, or peroneal nerves, resulting in an optimal functional outcome. One patient experienced postoperative thrombosis which leads to longer hospital stay, but no complications due to rotationplasty or nerve injuries were observed.

**Conclusion:**
An adequate visualization of the sciatic, tibial, and peroneal nerves was achieved by epineural indocyanine green injection which enabled a rapid and safe tumor resection and avoidance of an epineurectomy.

**Modified Borggreve-Van Nes-WinkelmannRotationplasty for Surgery in Developing Countries**
ID: 588

**Kategorie: DGOOC - Muskuloskeletale Tumoren – interdisziplinäre Versorgung**

Thiha Aung[1]

[1]*TH Deggendorf, Deggendorf, Deutschland*

**Background:**
Amputation is still the most common therapy for patients suffering from osteosarcoma in developing countries, despite the fact that limb salvage surgery e.g. Borggreve- Van Nes- Winkelmann rotationplasty for malignant tumors located within the distal femur or proximal tibia is the current state-of-the-art reconstructive procedure. A safe and reliable operation technique is crucial in order to perform a complex surgical procedure like the rotationplasty in third world countries with limited infrastructure and resources. The authors present seven cases of patients with osteosarcomas that received a Borggreve- Van Nes-Winkelmann rotationplasty with an evaluation of the procedures focusing on safety and sustainability.

**Materials and methods:**
From 2019 until 2020, seven young patients with osteosarcomas of the distal femur or proximal tibia were treated with Borggreve- Van Nes- Winkelmann rotationplasties in the Orthopaedic Hospital in Mandalay, Myanmar. As modification of the standard procedure the dissection and subsequent clamping of the femoral artery to minimize blood loss as well as the formation of an adipocutaneous flap that minimizes swelling and decreases the pressure on the vessels were successfully performed. This modified procedure resembles a safe and simplified surgical technique that is feasible under the circumstances of a developing country with good outcomes.

**Results:**
All patients showed good functional and aesthetic results. One of the seven patients needed secondary wound closure due to wound dehiscence.

**Conclusion:**
A simplified and safe operation technique for the performance of the Van Nes-Borggreve rotationplasty was adapted to the given constraints in developing countries and proved to be successful.
